# Carp Edema Virus and Cyprinid Herpesvirus-3 Coinfection is Associated with Mass Mortality of Koi (*Cyprinus carpio haematopterus*) in the Republic of Korea

**DOI:** 10.3390/pathogens9030222

**Published:** 2020-03-17

**Authors:** Sang Wha Kim, Sib Sankar Giri, Sang Guen Kim, Jun Kwon, Woo Taek Oh, Se Chang Park

**Affiliations:** Laboratory of Aquatic Biomedicine, College of Veterinary Medicine and Research Institute for Veterinary Science, Seoul National University, Seoul 08826, Korea

**Keywords:** carp edema virus, cyprinid herpesvirus-3, *Cyprinus carpio haematopterus*, mass mortality, coinfection

## Abstract

As koi and common carp gain importance in the Korean fish industry, the need for better diagnosis, prevention, and treatment of associated diseases has increased. In June 2019, the first known case of mass mortality involving cyprinid herpesvirus-3 (CyHV-3) and the second involving carp edema virus (CEV) occurred in a koi farm in Jeolla-do, Korea. Notably, the CEV exhibited a closer phylogenetic relationship with certain CEV strains originating from Poland, Germany, and India than with strains originating from China or Japan. Epidemiological studies and detailed surveillance and control for CEV and CyHV-3 are needed along with quarantine inspections.

## 1. Introduction

The global aquaculture production of koi and common carp has steadily increased since the 1950s and especially since the 1980s [1,2], rendering these species important for the Korean fish industry [3]. The need for better diagnosis, prevention, and treatment of diseases associated with these species increases as their value continues to increase. Among such diseases, the cyprinid herpesvirus-3 (CyHV-3) and carp edema virus (CEV) infections are gaining attention, as they manifest as subclinical infection and are associated with a high mortality rate; therefore, they pose a mounting threat to the industry. 

The CEV belongs to the family *Poxviridae*, subfamily *Chordopoxvirinae* [4]. CEV infection causes viral edema of carp, which was first reported in Japan in the 1970s [5,6]. Viral edema of carp is accompanied by clinical symptoms, including lethargy, anorexia, skin lesions, enophthalmos, edema, and gill necrosis [5,7]. Up to 80−100% of mortality occurs within 2−3 weeks of disease onset. 

The CEV has spread worldwide due to subclinical infections that are undetectable during disease screening. CEV outbreaks have been recently reported in numerous European and Asian countries [7,8,9,10]. In Korea, the first outbreak was reported in 2018; given that the identified CEV was more closely related to the CEV from Poland and the UK than that from the geographically closer Japan, there is an urgent need for epidemiological exploration of fish import routes and disease origins [8].

The CyHV-3 belongs to the family *Alloherpesviridae*, genus *Cyprinivirus*. This virus caused mass mortality in Israel and Germany in 1998 and in the UK in 1996 [11,12]. Cases of infected aquaculture and natural freshwaters were then reported in Europe, Asia, and Africa [13,14,15]. This spread was possible due to latent infections of CyHV-3 (as with CEV), which prevented efficient screening. CyHV-3 infection was listed as a World Organisation for Animal Health (OIE) reportable disease since 2006 due to the severity of the situation [16]. The first CyHV-3-infected case in Korea was reported in a koi broodstock in 2010 [17], and CyHV-3 has been included in the pathogen detection list for cultured juveniles for stock enhancement since 2009 in Korea [18]. 

CyHV-3 infection results in clinical signs, including lethargy, fatigue, gasping at the water surface, enophthalmos, gill necrosis, increased mucus secretion, and white patches on the body surface [19]. Mortality occurs 7−10 days after disease onset, and the mortality rate may be >90% within 2−3 weeks. The overall features and progression of the CEV and CyHV-3 infections are very similar; thus, it is almost impossible to differentiate between the two infections based on the clinical signs alone. Here, we report a case of mass mortality of koi associated with CEV and CyHV-3 coinfection. To the best of our knowledge, this is the first report of CEV and CyHV-3 coinfection in koi in Korea and the second report of CEV infection-related mass mortality of koi in Korea.

## 2. Materials and Methods 

### 2.1. Fish Sampling and Necropsy

In June 2019, a mass mortality event with an unknown cause of death was recorded at a koi farm in Jeolla-do, Korea. Hundreds of koi fish first exhibited lethargy and anorexia and then lay down on the tank floor, which eventually led to mass mortality. External examination was performed on the dead fish, and since their lesions were all similar, random sampling for further investigation was performed. Two fish were sampled and shipped to the Laboratory of Aquatic Biomedicine, Seoul National University, immediately after their death. The carcasses were maintained at 4 °C during shipping, and necropsy was performed immediately upon arrival. For convenience, the two koi fish were named koi-A (male) and koi-B (female). After gill clipping and skin scraping, the presence of external parasite infection was inspected with an optical microscope. For bacterial culture, the organ surface was sterilized using a heated blade and punctured to perform parenchymal swab sampling. Bacterial cultures of the hepatopancreas, gonad, spleen, and kidney were established for 48 h using tryptic soy agar plates at 24 °C. The kidney, hepatopancreas, spleen, gill, intestinal tissue, and muscle tissue were sampled and frozen for total DNA extraction and polymerase chain reaction (PCR). 

### 2.2. DNA Preparation and PCR Amplification

Total DNA was extracted from the kidney, hepatopancreas, spleen, gill, intestinal tissue, and muscle tissue of both fish using the DNeasy Blood & Tissue Kit (Qiagen, Hilden, Germany). PCR-restriction fragment length polymorphism (RFLP) was conducted to confirm subspecies according to Zhou et al. [20]. CyHV-3 and CEV infection were included in the differential diagnosis based on the history, clinical signs, and external examination and gross pathology results. PCR detection of the two viruses was performed. CEV was detected using two different PCR methods targeting the partial 4a protein gene, according to the protocol of Oyamatsu et al., and the 5′ untranslated region (UTR), according to the protocol of the Centre for Environment Fisheries and Aquaculture Science (CEFAS) Weymouth Laboratory, England (D. Stone, unpublished) [21]. PCR for CyHV-3 was performed, as previously described by Bercovier et al. for the thymidine kinase gene and by Gilad et al. for the genomic fragment 9/5 [22,23]. After CyHV-3 infection was confirmed, PCR was performed for detection of marker I and II, as described by Bigarre et al. in order to determine the lineage of the virus [24]. The sequence lengths of the PCR products were confirmed by gel electrophoresis using 1.7% agarose gel. The PCR products with targeted sequence sizes were gel-purified. The PCR methods of both Gilad et al. and Bercovier et al. provided much higher positive signal intensity for CyHV-3 in koi-A than in koi-B [22,23]. Accordingly, sequencing was performed using the PCR products from koi-A (Macrogen Korea, Seoul, Korea). Quantitative PCR (qPCR) for both pathogens were also performed following Adamek et al. and Gilad et al. [25,26]: 250 ng of total DNA was used for each qPCR reaction, and resulting Ct values were calibrated using koi glucokinase to match the number of host cells used for each assay in accordance with Holopainen et al. [27].

### 2.3. Cloning and Sequencing of the PCR Products

The PCR products were cloned using thymine and adenine (TA) cloning vector and sequenced (Macrogen Korea, Seoul, Korea). Since the sequences from koi-A and koi-B were 100% identical in the PCR products obtained using the protocols of both Oyamatsu et al. and CEFAS, all four TA cloning procedures were performed using the first-round PCR products from koi-A [21]. After cloning, sequencing was performed using universal primers, and all sequences were registered in the GenBank (GenBank accession number: MN545482−MN545485). 

### 2.4. Phylogenetic Relationship Analysis

A basic local alignment search tool (BLAST) search was performed using the sequences. Phylogenetic trees were drawn using previously registered sequences in the GenBank based on the BLAST results. Multiple alignments were performed using Clustal W (gap opening penalty: 15.00; gap extension penalty: 6.66), and neighbor-joining trees were drawn with MEGA-X version 10.0.5 [28,29]. The evolutionary distances were calculated with the Maximum Composite Likelihood method, and the trees were drawn to scale [30]. Missing data or gaps were deleted, and phylogeny was evaluated using 1000 bootstrap replicates.

## 3. Results and Discussion

**Figure 1:**
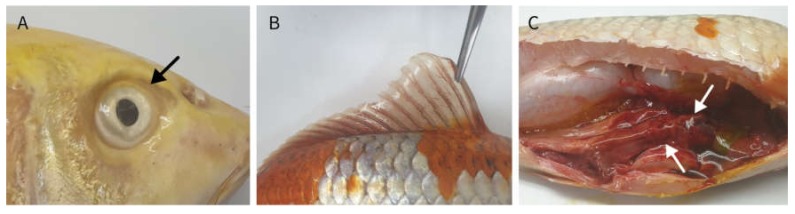
External examination and gross pathology of the koi: (**A**) enophthalmos of koi-A (black arrow), (**B**) hyperemia at the fin of koi-B, and (**C**) hemorrhagic hepatopancreas of koi-B (white arrows).

The average water temperature of the koi farm was 18.9 °C in June when the mass mortality occurred. This was in between seasons of late spring to early summer, recording average water temperature of above 18 °C for the first time in 2019. This is in line with the environmental conditions for both CEV and CyHV-3 infection, which occur when environmental temperature changes rapidly. Average fork length of the fish was 34 cm, and the koi group was presumed to be 2–3 years old according to Tempero et al. [31]. Both koi-A and koi-B exhibited enophthalmos; excessive mucus secretion; gill necrosis; hemorrhage in the gonads and hepatopancreas; and hyperemia at each fin base, the peri-anal region, and the abdominal body surface (Figure 1). Gill clipping and skin scraping did not reveal parasitic infections. The bacterial cultures of different organs did not reveal dominant colonies. Based on the premortem clinical signs and history, external examination results, gross pathology, and bacterial culture results, viral edema of carp was associated with the differential diagnosis of either CEV or CyHV-3 infection.

PCR-RFLP for subspecies identification indicated that the fish was *Cyprinus carpio haematopterus* [20]. PCR was performed with total DNA from the kidney, hepatopancreas, spleen, gill, and intestine. Both PCR methods for CEV detection revealed strong positive bands for the kidney, gill, and hepatopancreas DNA from both koi-A and koi-B. The detection method for CyHV-3 revealed positive bands for kidney, spleen, gill, and intestine DNA from koi-A and for kidney and gill DNA from koi-B. Overall, kidney and gill showed stronger band than others. The reason why the pathogens were not able to be detected by PCR in some samples is because the amount of the template DNA was not controlled by quantification or because the viral loads of some organ samples were too low to be detected by conventional PCR method. 

**Figure 2:**
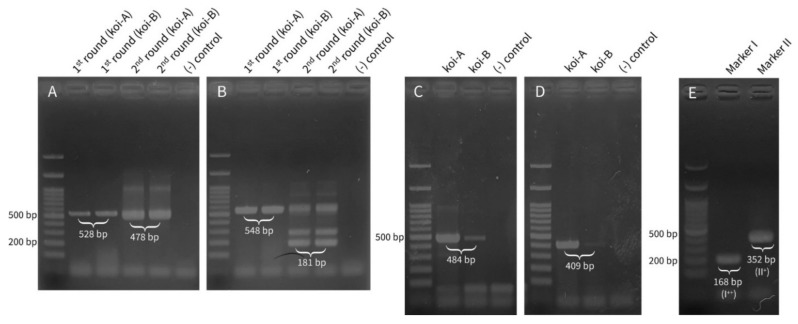
Gel electrophoresis of polymerase chain reaction (PCR) products: (**A**) PCR products of the 5ʹ untranslated region (Centre for Environment Fisheries and Aquaculture Science methods) for carp edema virus (CEV) detection using total DNA extracted from the gill. Both first- and second-round products of nested PCR are visualized. (**B**) Partial 4a protein gene PCR products for CEV detection using total DNA extracted from the gill [21]: Both first- and second-round products of nested PCR are visualized. (**C**) Genomic fragment 9/5 PCR products for cyprinid herpesvirus-3 (CyHV-3) detection [23]. (**D**) Partial thymidine kinase gene PCR products for CyHV-3 detection [22]. (**E**) Marker I and marker II PCR products for CyHV-3 lineage confirmation [24]. Since koi-A showed stronger positive signals in both Figure 2C,D, total DNA from the kidney of koi-A was used.

The gel electrophoresis results of the products of each PCR method for the organ-derived DNA are shown in Figure 2A–D. The gill total DNA was used for CEV detection, and kidney total DNA was used for CyHV-3 detection due to the corresponding higher viral load in these organs than in the other organs [32]. Although both methods for CEV detection used nested PCR, it was possible to confirm clear positive bands even after the first round of PCR (Figure 2A,B). Both koi fish exhibited positive bands, thus confirming the existence of CyHV-3; however, the signal intensities from koi-B were weaker than those from koi-A due to differences in the viral loads. 

By performing qPCR, CEV and CyHV-3 could be detected from all five organ samples of both koi-A and koi-B. Relative viral load in each organ was as follows: CEV: gill > intestine > kidney > hepatopancreas > spleen; CyHV-3: gill > kidney > intestine > spleen > hepatopancreas (Supplementary Table S1). This result matches the prior studies of viral load comparison in each disease [32,33]. In addition, overall viral loads of koi-B were lower than those of koi-A in both CyHV-3 and CEV. It suggests that koi-B died with smaller viral loads than koi-A. This may be determined by a variety of factors including differences in immunity, underlying diseases, nutritional conditions, and environmental situation. In any case, both CEV and CyHV-3 were detected in koi-A and koi-B and all indications, including the premortem behavioral changes and clinical signs, pathological signs, and PCR and qPCR results, suggested that the group of koi was infected with both CEV and CyHV-3. 

**Figure 3:**
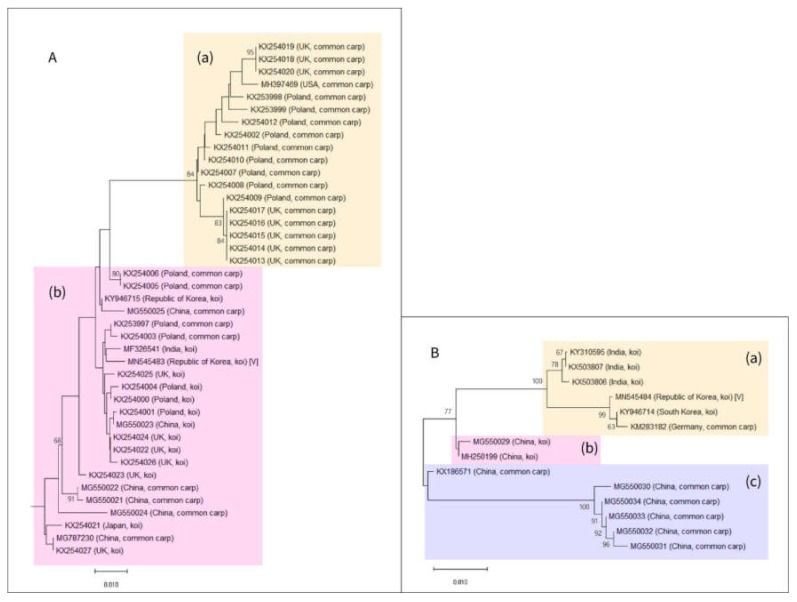
Phylogenetic tree of the carp edema virus (CEV) sequences: (**A**) Phylogenetic tree of the partial 4a protein gene sequences of CEV. The sequences are classified into genogroups I and II, according to Matras et al. [9]. (**A**)/(**a**) Genotype I. Percent identity to the query sequence on basic local alignment search tool (BLAST) analysis: 94.00–96.03%. (**A**)/(**b**) Genotype II. Percent identity to the query sequence on BLAST analysis: 96.93−99.16%. Both sequences derived from South Korea, including the one from this study (GenBank accession number: MN545483), belong to this group. (**B**) Phylogenetic tree of the 5ʹ untranslated region sequences for CEV: The sequences can be grouped into three clades. Scale bar: evolutionary distance in the unit of the number of base substitutions per site. (**B**)/(**a**) Clade (a) consists of both sequences reported from South Korea, including the one reported in this study (GenBank accession number: MN545484). (**B**)/(**b**) Clade (b) includes sequences derived from CEV-infected koi in China. (**B**)/(**c**) Clade (c) includes sequences derived from CEV-infected common carp in China.

The first-round PCR products for CEV detection were TA-cloned, sequenced, and subjected to a BLAST search, and a phylogenetic tree was drawn. With respect to the phylogenetic tree drawn using the PCR products obtained using the method of Oyamatsu et al., many additional sequences have been registered in the GenBank since the report by Matras et al. [9,21]. However, they are still clearly split into two groups (genogroups I and II) as classified by Matras et al. after the addition of the new sequences (Figure 3A) [9]. 

The CEV sequence detected in this study is included in genogroup II, the same group to which the previously reported CEV sequence from Korea (GenBank accession number: KY946715) belonged. The CEV from Korea was confirmed to be phylogenetically closer to certain strains of CEV from Poland and India than to those from the geographically closer Japan.

The phylogenetic tree based on the PCR products from the CEFAS method exhibited three different clades (Figure 3B). Clade (a) contained both CEV sequences reported from Korea, including the one from this study (GenBank accession number: MN545484). Moreover, this tree also indicates that the strains from Germany and India have a closer phylogenetic relationship with the strain from Korea than the strain from the geographically closer China. The closer phylogenetic relationship of CEV with strains from more distant locations emphasizes the need for an epidemiological investigation. Both clades (b) and (c) consisted of CEV sequences from China. In addition, clade (b) only contained koi-derived CEVs and clade (c) only contained common carp-derived sequences. Moreover, clades (b) and (c) differed from clade (a), which contained both koi- and common carp-derived sequences.

CyHV-3 PCR results were registered on GenBank with accession numbers of MN545482 and MN545485. MN545482 is a partial coding sequence of protein ORF89 (ORF89) and protein Allo37 (ORF90) genes, showing 100% identity with the sequences from Asian countries including Indonesia and Japan, followed by Poland, US, Israel, and China that are showing percent identity higher than 99% when BLAST searched. In the case of MN545485, a partial coding sequence of thymidine kinase gene, various countries including Mexico, China, Iraq, Iran, Indonesia, Poland, and Japan showed 100% identity on BLAST search. This sequence is thought to be a well-conserved part among CyHV-3 viruses from various regions and not a very efficient part to distinguish phylogeny.

The band sizes of the marker I and II PCR products were similar to the target sizes 168 bp and 352 bp, respectively (Figure 2E). Sequencing results of the PCR products confirmed the exact length and sequence of markers I and II. To conclude, the genotype of CyHV-3 in this study was confirmed as I^++^ II^+^, signifying that this virus belongs to the CyHV3-J lineage established by Aoki et al. [24,34]. Kim et al. identified two CyHV-3 genotypes in Korea: genotype I^++^ II^+^ from Pyeongtaek-si, Gyeonggi-do, and genotype I^−+^ II^−^ from Buan-gun, Jeollabuk-do [35]. In addition to genotype I^−+^ II^−^, which was identified in the Jeolla region, we demonstrated the presence of genotype I^++^ II^+^ (CyHV3-J) in this region I^++^ II^−^.

## 4. Conclusions

Here, we report the first incident of koi mass mortality associated with CyHV-3 and CEV coinfection in Korea. However, it is possible that many cases previously diagnosed as CyHV-3 infection (based on clinical symptoms and PCR tests) could have also involved CEV coinfection. A similar case was previously reported in China, and it is likely that coinfection has previously occurred but such cases have remained undetected [10]. Based on the high importance of koi and common carp in Korea, a detailed surveillance and control program for CEV and CyHV-3 is warranted. In addition, disease quarantine inspections on imported/exported fish should be performed with greater precision. The quarantine system, which has been limited to assessing the koi herpes virus, should be expanded to include CEV. Finally, research must be conducted on the treatment strategies for the disease.

## Supplementary Materials

The following are available online at https://www.mdpi.com/2076-0817/9/3/222/s1, Table S1: Ct values of qPCR for CEV and CyHV-3 detection.

## References

[1] FAO (2014). The State of World Fisheries and Aquaculture (SOFIA).

[2] FAO Fishery and Aquaculture Statistics. Global Production by Production Source 1950–2015 (FishstatJ). www.fao.org/fishery/statistics/software/fishstatj/en.

[3] Kim D., Kang J. (2012). Improvement of ornamental fish industry through analysis of recognition and market scale of the ornamental fish. J. Fish. Bus. Adm..

[4] Haenen O.L., Way M.K., Gorgoglione B., Ito T., Paley R., Bigarre L., Waltzek T. (2016). Novel viral infections threatening Cyprinid fish. Bull. Eur. Assoc. Fish Pathol..

[5] Murakami Y., Shitanaka M., Toshida S., Matsuzato T. (1976). Studies on mass mortality of juvenile carp: About mass mortality showing edema. Bull. Hiroshima Fresh Water Fish Exp. Stn..

[6] Ono S., Nagai A., Sugai N. (1986). A histopathological study on juvenile colorcarp, Cyprinus carpio, showing edema. Fish Pathol..

[7] Lewisch E., Gorgoglione B., Way K., El-Matbouli M. (2015). Carp edema virus/Koi sleepy disease: An emerging disease in Central-East Europe. Transbound. Emerg. Dis..

[8] Kim S.W., Jun J.W., Giri S.S., Chi C., Yun S., Kim H.J., Kim S.G., Kang J.W., Park S.C. (2018). First report of carp oedema virus infection of koi (Cyprinus carpio haematopterus) in the Republic of Korea. Transbound. Emerg. Dis..

[9] Matras M., Borzym E., Stone D., Way K., Stachnik M., Maj-Paluch J., Palusinska M., Reichert M. (2017). Carp edema virus in Polish aquaculture—Evidence of significant sequence divergence and a new lineage in common carp Cyprinus carpio (L.). J. Fish Dis..

[10] Ouyang P., Yang R., Chen J., Wang K., Geng Y., Lai W., Huang X., Chen D., Fang J., Chen Z. (2018). First detection of carp edema virus in association with cyprinid herpesvirus 3 in cultured ornamental koi, Cyprinus carpio L., in China. Aquaculture.

[11] Bretzinger A., Fischer-Scherl T., Oumouna M., Hoffmann R., Truyen U. (1999). Mass mortalities in koi carp, Cyprinus carpio, associated with gill and skin disease. Bull. Eur. Assoc. Fish Pathol..

[12] Perelberg A., Smirnov M., Hutoran M., Diamant A., Bejerano Y., Kotler M. (2003). Epidemilogical Description of a New Viral Disease Afflicting Cultured Cyprinus Carpio In Israel. Isr. J. Aquac..

[13] Haenen O.L., Way M.K., Bergmann S.M., Areil E. (2004). The emergence of koi herpesvirus and its significance to European aquaculture. Bull. Eur. Assoc. Fish Pathol..

[14] Sano M., Ito T., Kurita J., Yanai T., Watanabe N., Miwa S., Iida T. (2004). First detection of koi herpesvirus in cultured common carp Cyprinus carpio in Japan. Fish Pathol..

[15] Kurita J., Yuasa K., Ito T., Sano M., Hedrick R.P., Engelsma M.Y., Haenen O.L.M., Sunarto A., Kholidin E.B., Chou H. (2009). Molecular epidemiology of koi herpesvirus. Fish Pathol..

[16] Taylor N.G.H., Dixon P.F., Jeffery K.R., Peeler E.J., Denham K.L., Way K. (2010). Koi herpesvirus: Distribution and prospects for control in England and Wales. J. Fish Dis..

[17] Gomez D.K., Joh S.J., Jang H., Shin S.P., Choresca C.H., Han J.E., Kim J.H., Jun J.W., Park S.C. (2011). Detection of koi herpesvirus (KHV) from koi (Cyprinus carpio koi) broodstock in South Korea. Aquaculture.

[18] Cho M.Y., Won K.M., Han H., Kim H.J., Jee B., Kim S., Lee S.J., Kim J.W., Park M.A. (2013). Current status of detection of aquatic animal pathogens in cultured juveniles for stock enhancement from 2009 to 2012. J. Fish Pathol..

[19] Hedrick R.P., Gilad O., Yun S.C., Mcdowell T.S., Waltzek T.B., Kelley G.O., Adkison M.A. (2005). Initial isolation and characterization of a herpes-like virus (KHV) from koi and common carp. Bull. Fish. Res. Agency.

[20] Zhou J., Wang Z., YE Y., Wu Q. (2003). PCR-RFLP analysis of mitochondrial DNA ND5/6 region among 3 subspecies of common carp (Cyprinus carpio L.) and its application to genetic discrimination of subspecies. Chin. Sci. Bull..

[21] Oyamatsu T., Matoyama H., Yamamoto K., Fukuda H. (1997). A trial for the Detection of Carp Edema Virus by Using Polymerase Chain Reaction. Aquac. Sci..

[22] Bercovier H., Fishman Y., Nahary R., Sinai S., Zlotkin A., Eyngor M., Gilad O., Eldar A., Hedrick R.P. (2005). Cloning of the koi herpesvirus (KHV) gene encoding thymidine kinase and its use for a highly sensitive PCR based diagnosis. BMC Microbiol..

[23] Gilad O., Yun S., Andree K.B., Adkison M.A., Zlotkin A., Bercovier H., Eldar A., Hedrick R.P. (2002). Initial characteristics of koi herpesvirus and development of a polymerase chain reaction assay to detect the virus in koi, Cyprinus carpio koi. Dis. Aquat. Org..

[24] Bigarre L., Baud M., Cabon J., Antychowicz J., Bergmann S.M., Engelsma M., Pozet F., Reichert M., Castric J. (2009). Differentiation between Cyprinid herpesvirus type-3 lineages using duplex PCR. J. Virol. Methods.

[25] Adamek M., Jung-Schroers V., Hellmann J., Teitge F., Bergmann S.M., Runge M., Kleingeld D.W., Way K., Stone D.M., Steinhagen D. (2016). Concentration of carp edema virus (CEV) DNA in koi tissues affected by koi sleepy disease (KSD). Dis. Aquat. Org..

[26] Gilad O., Yun S., Zagmutt-Vergara F.J., Leutenegger C.M., Bercovier H., Hedrick R.P. (2004). Concentrations of a Koi herpesvirus (KHV) in tissues of experimentally infected *Cyprinus carpio koi* as assessed by real-time TaqMan PCR. Dis. Aquat. Org..

[27] Holopainen R., Honkanen J., Jensen B.B., Ariel E., Tapiovaara H. (2011). Quantitation of ranaviruses in cell culture and tissue samples. J. Virol. Methods.

[28] Saitou N., Nei M. (1987). The neighbor-joining method: A new method for reconstructing phylogenetic trees. Mol. Biol. Evol..

[29] Kumar S., Stecher G., Li M., Knyaz C., Tamura K. (2018). MEGA X: Molecular Evolutionary Genetics Analysis across computing platforms. Mol. Biol. Evol..

[30] Tamura K., Nei M., Kumar S. (2004). Prospects for inferring very large phylogenies by using the neighbor-joining method. Proc. Natl. Acad. Sci. USA.

[31] Tempero G.W., Ling N., Hicks B.J., Osborne M.W. (2010). Age composition, growth, and reproduction of koi carp (*Cyprinus carpio*) in the lower Waikato region, New Zealand. N. Zeal. J. Mar. Fresh..

[32] Adamek M., Oschilewski A., Wohlsein P., Jung-Schroers V., Teitge F., Dawson A., Gela D., Piackova V., Kocour M., Adamek J. (2017). Experimental infections of different carp strains with the carp edema virus (CEV) give insights into the infection biology of the virus and indicate possible solutions to problems caused by koi sleepy disease (KSD) in carp aquaculture. Vet. Res..

[33] World Organisation for Animal Health (OIE) (2019). Chapter 2.3.7. Infection with Koi Herpesvirus. Manual of Diagnostic Tests for Aquatic Animals.

[34] Aoki T., Hirono I., Kurokawa K., Fukuda H., Nahary R., Eldar A., Davison A.J., Waltzek T.B., Bercovier H., Hedrick R.P. (2007). Genome sequences of three koi herpesvirus isolates representing the expanding distribution of an emerging disease threatening koi and common carp worldwide. Am. Soc. Microbiol..

[35] Kim H.J., Kwon S.R. (2013). Evidence for two koi herpesvirus (KHV) genotypes in South Korea. Dis. Aquat. Org..

