# Supplementary material for: Carp Edema Virus and Cyprinid Herpesvirus-3 Coinfection is Associated with Mass Mortality of Koi (Cyprinus carpio haematopterus) in the Republic of Korea
Source: Pathogens. 2020 Mar 17;9(3):222. doi: 10.3390/pathogens9030222 (PMC7157718; doi:10.3390/pathogens9030222)
Supplement: Supplementary file 1 [file pathogens-09-00222-s001.pdf]

Supplementary Materials

Table S1. Ct values of qPCR for CEV and CyHV-3 detection.

Organ	Koi ID	Ct Value	
		CyHV-3	CEV
Kidney	Koi-A	18.86	36.66
	Koi-B	33.62	27.95
	Average	26.24	32.31
Hepatopancreas	Koi-A	33.66	28.92
	Koi-B	35.76	39.93
	Average	34.71	34.42
Spleen	Koi-A	31.21	34.50
	Koi-B	36.46	37.25
	Average	33.84	35.87
Gill	Koi-A	21.97	19.85
	Koi-B	27.75	20.92
	Average	24.86	20.38
Intestine	Koi-A	29.19	29.85
	Koi-B	31.91	33.05
	Average	30.55	31.45
Master Mix negative control	Koi-A	>40	>40
	Koi-B	>40	>40
	Average	>40	>40
Template DNA negative control	Koi-A	>40	>40
	Koi-B	>40	>40
	Average	>40	>40

Ct values are calibrated with Ct values of koi glucokinase to match the number of host cells used for each assay [27]. Average value is the average of koi-A and koi-B of each assay.
